# ProteinVR: Web-based molecular visualization in virtual reality

**DOI:** 10.1371/journal.pcbi.1007747

**Published:** 2020-03-31

**Authors:** Kevin C. Cassidy, Jan Šefčík, Yogindra Raghav, Alexander Chang, Jacob D. Durrant

**Affiliations:** 1 Department of Biological Sciences, University of Pittsburgh, Pittsburgh, Pennsylvania, United States of America; 2 Faculty of Information Technology, Czech Technical University in Prague, Prague, Czech Republic; Hebrew University of Jerusalem, ISRAEL

## Abstract

Protein structure determines biological function. Accurately conceptualizing 3D protein/ligand structures is thus vital to scientific research and education. Virtual reality (VR) enables protein visualization in stereoscopic 3D, but many VR molecular-visualization programs are expensive and challenging to use; work only on specific VR headsets; rely on complicated model-preparation software; and/or require the user to install separate programs or plugins. Here we introduce ProteinVR, a web-based application that works on various VR setups and operating systems. ProteinVR displays molecular structures within 3D environments that give useful biological context and allow users to situate themselves in 3D space. Our web-based implementation is ideal for hypothesis generation and education in research and large-classroom settings. We release ProteinVR under the open-source BSD-3-Clause license. A copy of the program is available free of charge from http://durrantlab.com/protein-vr/, and a working version can be accessed at http://durrantlab.com/pvr/.

## Author summary

Proteins are microscopic machines that help maintain, defend, and regulate cells. Properly understanding the three-dimensional structures of these machines–as well as the small molecules that interact with them–can advance scientific fields ranging from basic molecular biology to drug discovery. Virtual reality (VR) is a powerful tool for studying protein structures. But many current systems for viewing molecules in VR, though effective, have challenging usability limitations. We have created a new web application called ProteinVR that overcomes these challenges. ProteinVR enables VR molecular visualization in users’ browsers, without requiring them to install a separate program or plugin. It runs on a broad range of desktop, laptop, and mobile devices. For users without VR headsets, ProteinVR leverages mobile-device orientation sensors or video-game-style keyboard navigation to provide an immersive experience. We release ProteinVR as open-source software and have posted a working version at http://durrantlab.com/pvr/.

This is a *PLOS Computational Biology* Software paper.

## Introduction

Molecular visualization can provide structural, biological, and pharmacological insights that cannot be obtained in any other way. Traditionally, researchers and educators have used 2D schema (e.g., images in a scientific article or textbook) to represent molecular structures [1]. Carefully shaded 2D images can convey some 3D information, but the depth, size, and placement of structural motifs are missing, making it difficult to accurately discern protein active sites, binding domains, and functions. Standard molecular-visualization computer programs, e.g., VMD [2] and PyMOL [3], improve 3D understanding by projecting 3D models onto 2D screens, allowing users to rotate and examine them in a faux 3D environment [2–6]. But these visualizations are still only approximations of the true 3D structures—pictures of things, rather than the things themselves [7]. Perceiving the spatial arrangements of interacting moieties continues to be challenging in some contexts.

Virtual reality (VR) overcomes these limitations by displaying models in stereoscopic 3D. Users are thus able to better perceive the spatial arrangements of protein binding pockets and protein/ligand interactions [8–13]. Compared to traditional visualization approaches, VR 1) provides a wider field of view, 2) allows users to observe molecules from within without requiring the use of clip planes, and 3) requires only head movements to change the viewpoint [7, 10].

In the past, the high cost of VR hindered efforts to popularize true 3D visualization systems (i.e., systems that deliver different images to each eye) [14, 15]. But with recent advances, low-end VR headsets now work with popular smartphones and are available for under $10, making VR technology broadly accessible [16]. Even high-end headsets that allow users to walk about the room in a simulated 3D environment cost less than $500.

We here present ProteinVR, a new open-source system that leverages these recent advances in VR to better visualize proteins and protein/ligand complexes (S1 Video). ProteinVR is unique among VR-based molecular-visualization programs in that it is entirely web based. Modern web browsers run on many operating systems and computing devices, so web-based visualization systems are immediately accessible on a broad range of desktops, laptops, and mobile devices, without requiring the installation of any third-party programs or plugins. ProteinVR also supports a number of low- and high-end VR headsets. Where VR is not available, it leverages mobile-device orientation sensors or video-game-style keyboard navigation to provide users with as engaging and immersive an experience as possible.

ProteinVR will be a useful tool for both the research and educational community. It allows researchers to better examine molecular structures and to collaboratively share molecular visualizations via convenient public URLs. It also allows educators and outreach-program coordinators to easily share instructive 3D scenes with students and the broader public. We release ProteinVR under the terms of the open-source BSD-3-Clause license. A copy is available free of charge from http://durrantlab.com/protein-vr/, and a working version of the app can be accessed at http://durrantlab.com/pvr/.

## Design and implementation

### ProteinVR viewer

**Fig 1:**
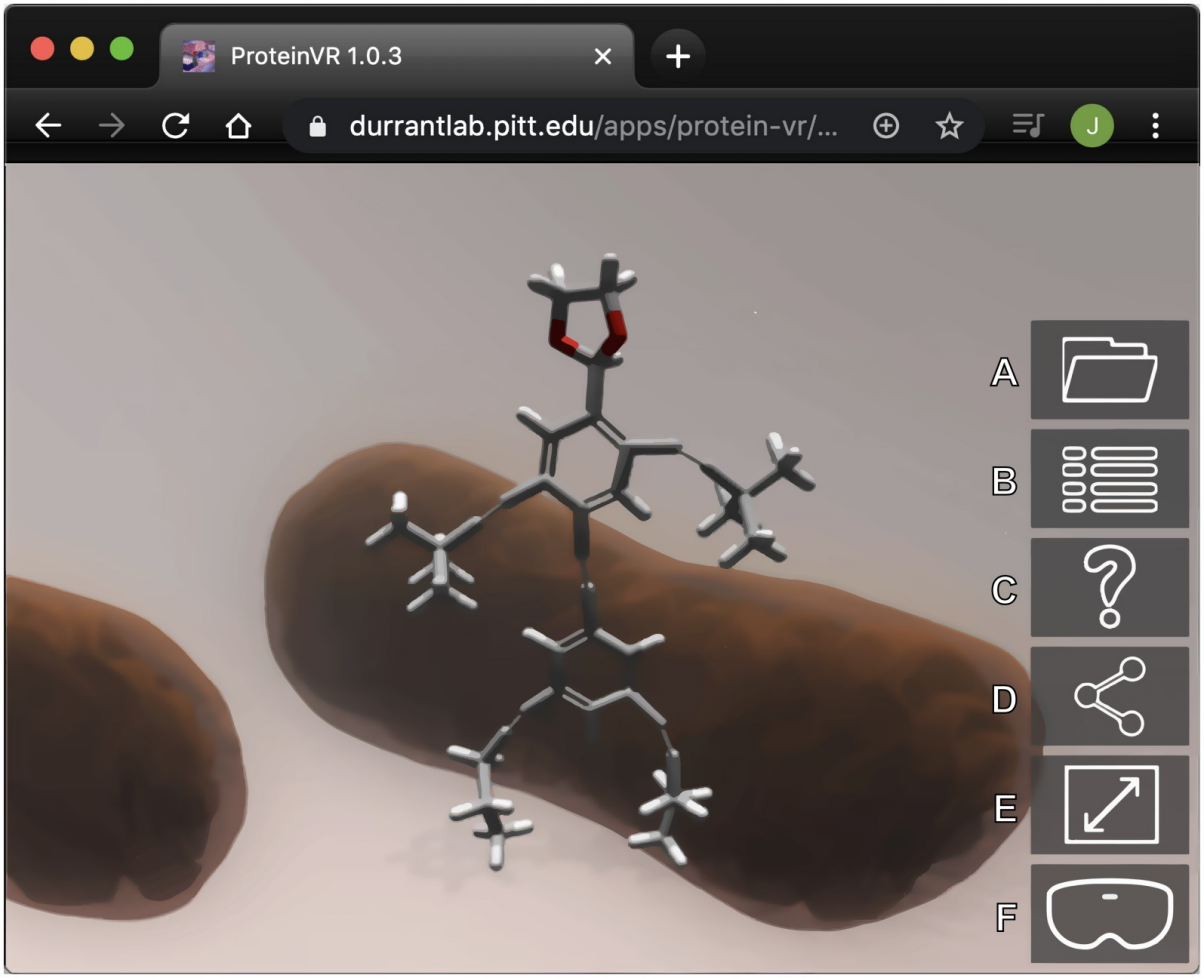
An illustration of a ProteinVR scene with the default NanoKid molecule visualized. The molecule is positioned within a skybox. Several buttons are available from the main screen. A) Load a new molecule and environment. B) Open the 2D menu. C) Provide help. D) Enter follow-the-leader mode. E) View in full screen. F) Enter VR mode.

ProteinVR leverages new and emerging technologies such as 3D web graphics and VR headsets to display relationships between molecular components in full 3D (Fig 1 and S1 Fig). It is written in TypeScript, a programming language that can be compiled to JavaScript for use in web browsers. The WebVR JavaScript application programming interface (API) allows ProteinVR to access the hardware necessary to render content in VR (e.g., the graphical processing unit, connected VR headsets, etc.). To date, WebVR is supported on a number of browsers, e.g., Chrome (Android), Firefox (Windows), and Oculus Browser. Given the rising popularity of VR, support will certainly broaden in the coming years.

ProteinVR uses 3Dmol.js [5] to generate molecular models for VR viewing. 3Dmol.js is a JavaScript library that displays molecular structures on a 2D HTML canvas. To do so it must store those structures as 3D models in memory. Importantly, 3Dmol.js includes the ability to export these internal models in the Virtual Reality Modeling Language (VRML) format [17]. Though newer formats have superseded VRML [18], we use VRML only to enable internal communication between libraries, so the user need not be concerned with these details. ProteinVR hides 3Dmol.js in the background and instead reads the 3D VRML data directly. It uses this data to render the molecular models within its own VR viewport. This approach enables quick, on-the-fly molecular-style changes involving any of the many representations available through 3Dmol.js (e.g., cartoon, stick, surface, etc.).

### 3D environments

To provide context and to orient the user in 3D space, ProteinVR positions molecular models within 3D environments (Fig 1). We used Blender, an open-source 3D modeling program, to generate these environments. Blender’s Cycles renderer is particularly useful for pre-calculating the photorealistic shadows and textures associated with unmoving/unchanging objects. Pre-rendering environments improves performance in the browser. Rather than calculate all environment shadows, ProteinVR needs only (optionally) calculate the shadows cast by the molecular models themselves.

To accommodate a broad range of devices, several simple default environments are included in the ProteinVR download. These environments ensure that ProteinVR can operate “out of the box” on even low-powered (e.g., mobile-phone-based) devices and VR headsets. The scenes consist of only a skybox (i.e., a large cube that surrounds the virtual camera, regardless of its position). Two-dimensional pictures of distant objects are projected onto the cube faces to suggest a larger surrounding environment. ProteinVR includes skybox-based environments that depict the inside of a blood vessel, the lipid-bilayer surface of a cell, and the intracellular space (Fig 1). For cases where none of these environments are biologically appropriate, users can also select two different “blank” skyboxes containing only decorative clouds.

## Results

### Advantages of web-based VR

ProteinVR’s web-based approach has many advantages. First, ProteinVR uses native web technologies, so it does not require users to download or install any programs or plugins beyond the web browser itself. In this sense it differs from other VR molecular-visualization programs [7, 8, 10, 11, 19–22], which require users to download a stand-alone program that may not be compatible with all operating systems. Aside from easing scientific collaboration, this installation-free approach is particularly helpful in educational settings, where expecting students to install a separate program is impractical. Sharing ProteinVR molecular scenes is as simple as sending collaborators or students a convenient URL.

Second, our web-based approach ensures broad compatibility. ProteinVR is built using BabylonJS, a free, open-source game engine compatible with all modern web browsers. Such web browsers are available on all major platforms, including various operating systems (e.g., Windows, macOS, Linux, Android, iOS). BabylonJS also ensures compatibility with a broad range of computer-hardware setups (e.g., laptop/desktop computers, mobile devices, VR headsets). Where VR is available, ProteinVR uses BabylonJS to leverage many different kinds of VR headsets. Where VR is not available, ProteinVR uses other hardware (e.g., an orientation sensor, a 2D monitor, etc.) to provide as immersive an experience as possible.

Beyond ensuring broad compatibility, BabylonJS also helps future-proof ProteinVR. ProteinVR uses BabylonJS to access the underlying WebVR API. This API is subject to ongoing changes—so much so that it may soon be replaced by an entirely new API (WebXR) that additionally enables augmented reality (AR). The BabylonJS community actively updates the game-engine code so that BabylonJS-powered software such as ProteinVR need not be entirely rewritten every time the API changes.

### Basic usage

When users first open ProteinVR, the application displays the default molecule NanoKid [23] (Fig 1). After a few seconds, a simple popup form appears where users can type the PDB ID or URL of the molecular model they wish to visualize. The same form also allows users to indicate the 3D environment in which to place the molecular model, as well as whether the molecule should cast shadows. After clicking the “Load Molecule” button, NanoKid is replaced with the desired molecular structure.

**Fig 2:**
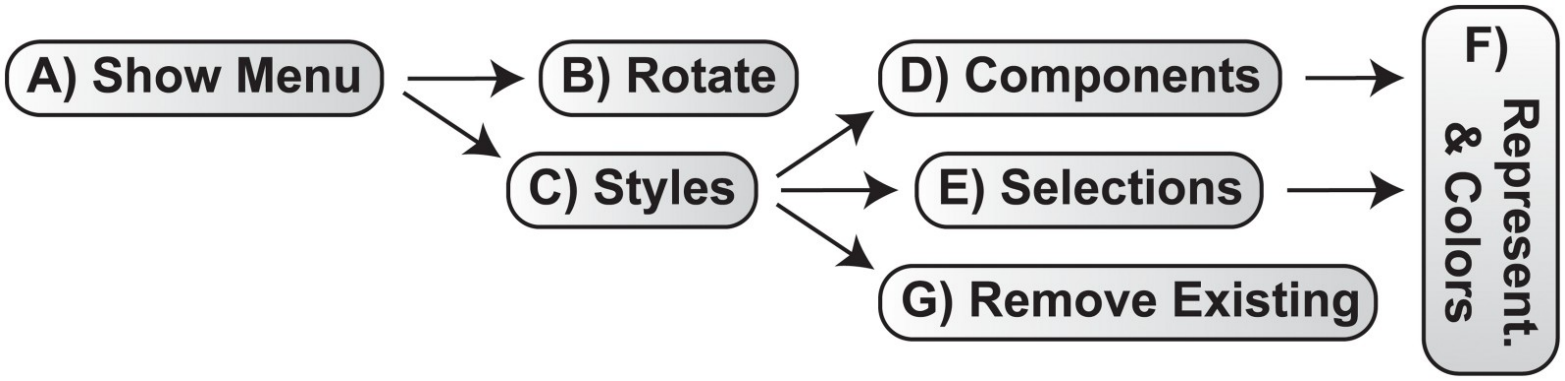
A schematic of the ProteinVR menu. A) Open the menu system using either the 2D or 3D button. B) Access the “Rotate” submenu. C) Access the “Styles” submenu. D) Change the style of common, pre-defined molecular components. E) Change the style of selected atoms specific to the loaded molecule itself. F) Change the representation and/or color of the selected atoms/components. G) Remove previously specified styles.

Several 2D buttons appear on the right of the screen that are only accessible when not in VR mode (Fig 1A–1F). The first allows users to load a new molecule/environment; the second opens a menu for changing the molecular style and rotation (Fig 2); the third provides a useful help system; the fourth generates a sharable URL that others can use to mirror the ProteinVR scene on their own devices (see “Leader Mode” below); and the fifth and sixth put ProteinVR into full-screen and VR mode, respectively.

The same menu for changing the molecular style and rotation (Fig 1B) is also accessible from within VR (Fig 2 and S1 Video). ProteinVR places a 3D button at the user’s feet with the text “Show Menu” (Fig 2A). Clicking the button opens a 3D version of the menu, embedded in the VR scene itself. Users on laptop/desktop computers can click the button using a mouse or keyboard (space bar); users on mobile devices without VR headsets can simply tap their screens; and users with VR headsets can pull the VR-headset or VR-controller trigger button.

At the top-most level, the ProteinVR menu is divided into two broad categories (Fig 2B and 2C). The “Rotate” submenu allows users to rotate the molecule about the X, Y, or Z axis. The “Styles” submenu contains further submenus that allow users to change how the molecule is displayed, both in terms of the molecular representation (e.g., cartoon, sphere, stick, surface) and the color (e.g., white, color by element, etc.) (Fig 2D–2F). “Styles > Components” applies these changes to common, pre-defined molecular components (e.g., proteins, ligands, nucleic acids, water molecules). “Styles > Selections” applies changes to the model using characteristics specific to the loaded molecule itself (e.g., specific residues, elements, chains, etc.). And “Styles > Remove Existing” allows users to remove previously specified representations/colors (Fig 2G).

ProteinVR also makes it easy to save molecular scenes with custom visualizations such as these. Every time a molecular representation is loaded, rotated, or otherwise altered, ProteinVR updates the browser URL to track the change. Copying the URL at any point into a new browser tab–even on a different device–recreates the exact same ProteinVR scene.

Interested readers may wish to view S1 Video, which illustrates many of the ProteinVR features described in this section.

### Display modes

To accommodate a broad range of devices, ProteinVR runs in four modes: VR mode, device-orientation mode, desktop mode, and leader mode. In all four, ProteinVR uses video-game-style navigation. Objects reside at fixed positions in a 3D environment, and the camera moves (or teleports) to different locations in the scene.

#### VR mode

VR mode is ideal when users have access to a VR headset (e.g., an Oculus Rift, Oculus Quest, Oculus Go, HTC Vive, or Google-Cardboard compatible viewer). This mode provides a fully immersive experience wherein users can view their molecular structures in stereoscopic 3D. The 3D environments are particularly useful in VR mode, as they improve the sense of immersion. By allowing viewers to orient themselves spatially, 3D environments may also reduce VR sickness [24], which is thought to result from a perceived disconnect between the 3D scene presented to the eyes and the movement/orientation of the head. To enter VR mode, users must first attach a VR headset as well as any hand controllers. They then click the VR button in the main ProteinVR screen (Fig 1F).

**Fig 3:**
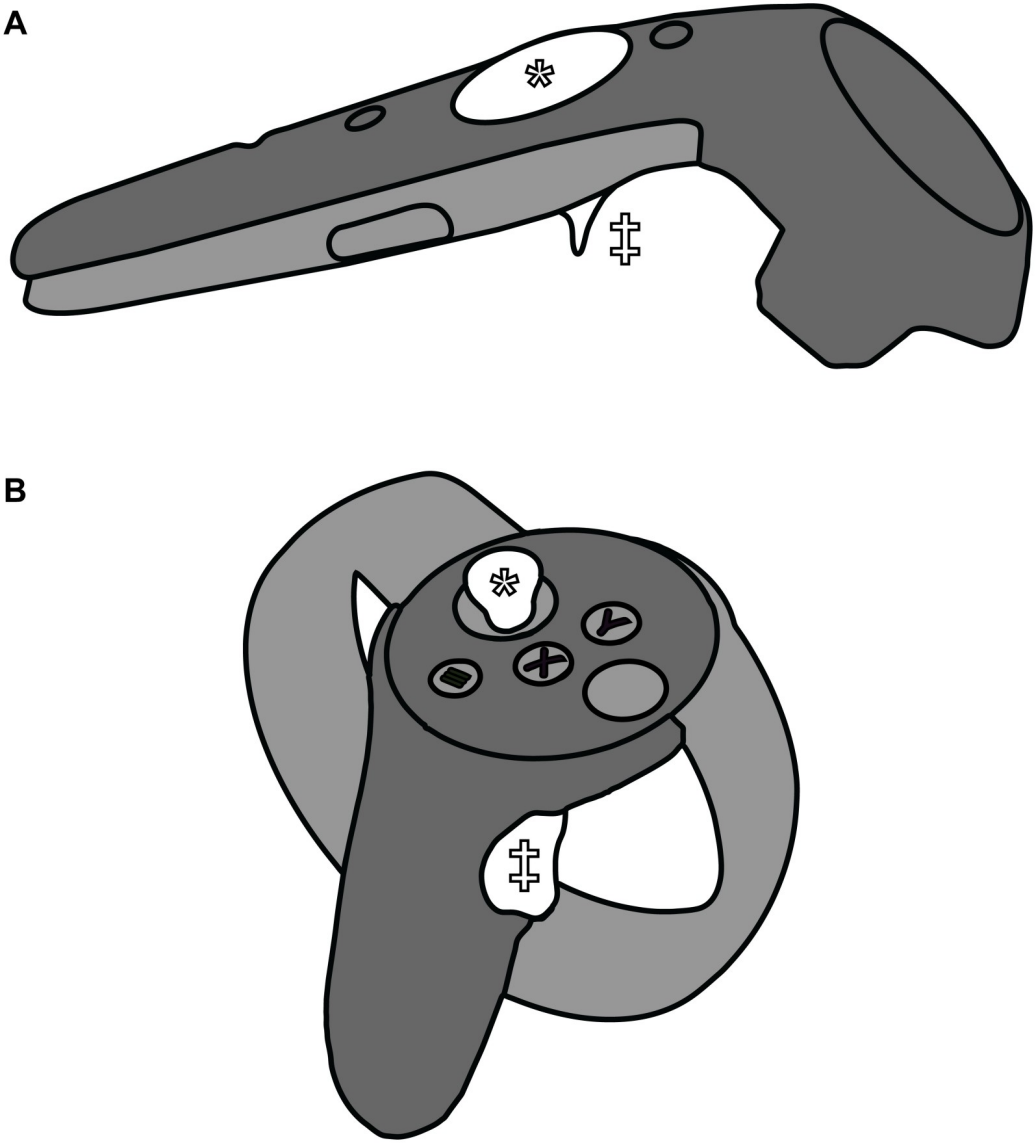
Illustrations of the controller buttons that enable navigation in VR mode. Different VR controllers have different physical configurations, but most include a trigger button (marked with a double dagger) and a trackpad or joystick (marked with an asterisk). A) HTC Vive controller (model 2PR7100). B) Oculus Touch controller (first generation).

In VR mode, users can look about the scene by physically moving their heads. Some VR headsets also allow users to navigate to nearby locations by physically moving about the room. Teleportation navigation enables movement to distant points in the virtual world (S1 Video). A simple navigation sphere indicates the current teleport destination. When using a VR headset that lacks hand controllers (e.g., Google Cardboard), this sphere appears on the object immediately in front of the user’s gaze. When using a headset with hand controllers (e.g., the HTC Vive, Oculus Rift, Oculus Quest, or Oculus Go), the sphere appears at the location where the user is pointing. To teleport to the location of the sphere, the user simply presses the VR-headset button, the VR-controller trigger (Fig 3A), the keyboard space bar, or the mouse click button.

VR controllers with trackpads or joysticks enable more fine-grained movements (Fig 3B). To slowly move forward or backward in the direction of the navigation sphere, users can simply press up or down on the trackpad/joystick. To rotate without having to rotate the head (e.g., to reset the view), users can press left or right.

We have specifically tested VR-mode on the operating-system, web-browser, and VR-headset setups indicated in Table 1. In some cases, it was necessary to explicitly enable the WebVR API and/or browser access to the device-orientation sensors. VR technology is rapidly evolving; a web search can reveal the steps necessary (if any) to fully enable VR in a given browser of choice.

**Table 1:** ProteinVR compatibility.

OS	Browser	VR	Orientation	Desktop	Leader
Windows	Chrome 77.0.3865	No	—	Yes	Desktop
Windows	Firefox 69.0.1	Vive / Rift	—	Yes	Vive / Rift / Desktop
macOS	Chrome 78.0.3904	—	—	Yes	Desktop
macOS	Firefox 68.0.2	—	—	Yes	Desktop
macOS	Safari 12	—	—	Yes	No
Linux	Chromium 77.0.3865	—	—	Yes	Desktop
Linux	Firefox 69.0.1	—	—	Yes	Desktop
Android	Chrome 77.0.3865	Cardboard	Yes	—	Cardboard / Orientation
Android	Firefox 68.1.1	Cardboard	Yes	—	Cardboard / Orientation
iOS	Safari 12 (PWA)	Cardboard	Yes	—	No
Oculus Quest	Oculus Browser 7.1.3	Yes	—	—	Yes
Oculus Go	Oculus Browser 7.0.13	Yes	—	—	Yes

We have tested ProteinVR on several operating-system/web-browser/VR-headset combinations. Operating systems (OS) include Windows (Windows 10 Pro 1809), macOS (macOS Mojave 10.14.5), Linux (Ubuntu Linux 18.04.2 LTS), Android (Android 9), iOS (iOS 12.4.1), Oculus Quest (Android 7.1.1), and Oculus Go (Android 8.0.0). “—” indicates not applicable or not tested. PWA stands for progressive web application. Select hardware specifications are listed in S1 Table.

*Desktop computers*.

We have verified that VR mode works well on Windows 10. We currently recommend the Firefox web browser. We have struggled to enable VR on Windows 10 Google Chrome, though we expect continued improvements in future versions given the expanding popularity of VR. Fortunately, Firefox provides a stable WebVR implementation that is enabled by default.

VR support in macOS is currently limited, though Apple has plans to expand support in the future. In preliminary tests, we did once manage to get WebVR working (albeit with a very low frame rate) on an HTC Vive connected to a MacBook Pro with an external graphics card. But we have not been able to reproduce that preliminary success and so cannot currently recommend macOS for VR.

*Mobile devices*.

VR mode also works well on most mobile devices. The WebVR API on Android is easy to access. In contrast, WebVR access on iOS is currently challenging. iOS mobile Safari does not allow webpages to hide the browser address bar, as required for VR visualization using mobile (e.g., Google Cardboard) headsets. Additionally, iOS does not allow the mobile Safari browser to access the device’s orientation sensors by default, making it impossible for ProteinVR to respond to head movements. Apple requires all third-party browsers on iOS (e.g., Chrome, Firefox) to use the same WebKit framework and JavaScript engine that Safari does, so it is not currently possible to overcome these challenges by switching to another browser.

To eliminate the address bar on iOS, users should install ProteinVR as a progressive web app (PWA). PWA installation places a ProteinVR icon on the device’s home screen and allows ProteinVR to run in full-screen mode. Simply visit the ProteinVR website via mobile Safari and use the browser’s “Share > Add to Home Screen” menu item. Additionally, users must enable access to the device-orientation sensors (even if running ProteinVR as a PWA) via Settings > Safari > Motion & Orientation Access. We are hopeful that Apple will simplify this process in the future as it expands its VR support.

*VR controllers*.

We have found that WebVR occasionally fails to recognize connected VR controllers. Users who struggle with the controllers may find the following tips helpful:

1. Turn on the controllers *before* entering VR.
2. On VR systems with multiple controllers (e.g., one for each hand), turn on all controllers, even though teleportation navigation requires only one.
3. When using the Oculus Go headset (Oculus Browser), enter VR mode, press the Oculus button, and select the “Resume” menu item to force controller recognition.

#### Device-orientation mode

Device-orientation mode is ideal when viewing ProteinVR scenes on mobile devices with orientation sensors. If ProteinVR detects such sensors, it automatically updates its viewport to match the orientation of the device itself. Users can thus view their molecular structures from different angles by physically reorienting their devices. ProteinVR also uses teleportation navigation in device-orientation mode. A navigation sphere (placed in the direction the mobile device is pointing) indicates the current teleport destination. To teleport to the location of the sphere, the user simply taps on the mobile-device screen.

We have specifically tested device-orientation mode on the operating-system/web-browser combinations indicated in Table 1. In our experience, Google Chrome on Android provides the easiest device-orientation experience. On iOS, users must explicitly enable access to the device-orientation sensors via Settings > Safari > Motion & Orientation Access.

#### Desktop mode

If neither a VR headset nor an orientation sensor is available, ProteinVR runs in desktop mode. Desktop mode uses a standard keyboard-and-mouse navigation system similar to that commonly used in video games. The arrow keys (or WASD keys) move forward, backward, and sideways. Clicking and dragging with the mouse changes the viewing angle. If the user clicks on the full-screen button in the main window (Fig 1E), ProteinVR instead changes the viewing angle whenever the mouse moves, without requiring an accompanying click. Teleportation navigation is also available for those who wish to use it. To teleport to the navigation sphere, the user need only press the space bar.

We have specifically tested desktop mode on the operating-system/web-browser combinations indicated in Table 1. As desktop mode uses only well-established web technologies, it runs on virtually any modern desktop browser.

#### Leader mode

Finally, ProteinVR can run in “leader mode.” This mode transforms the program into a powerful presentation tool. In many scenarios, multiple users may wish to visualize the same ProteinVR scene together. For example, a presenter may wish to use VR navigation to illustrate a specific molecular structure while simultaneously projecting the same view onto the screen behind her. Similarly, a teacher may wish to visualize a specific protein/ligand interaction using an advanced VR headset (e.g., the Oculus Rift) while his students view the same interaction on their phones.

A technology called WebRTC enables direct communication between leader and follower instances. When running in “leader” mode, ProteinVR broadcasts the user’s location in the 3D scene, as well as information about how the molecule of interest is currently represented. Broadcasting is available from VR headsets, Android phones, laptops, and desktops (Table 1). Safari and iOS are not currently supported. In contrast, when running in “follower” mode, ProteinVR receives this information from the designated leader and automatically updates the scene to match whatever the leader is currently seeing. Only 2D (desktop-mode-style) viewing is available in follower mode because VR viewing-angle updates independent of head movements may cause VR sickness [24].

### Examples of use

It is challenging to fully grasp the advantages of VR without entering the virtual world. Fortunately, the ProteinVR web app is easily accessible (http://durrantlab.com/pvr/). We encourage users to explore the app directly to better appreciate the benefits of our approach.

Though descriptions and figures cannot do full justice to the VR experience, we nevertheless describe two ProteinVR test cases. These examples illustrate how our VR implementation provides insights that are difficult to obtain via traditional, non-VR methods.

#### Small-molecule docking poses

First, we considered applications to rational ligand design. We examined *T. brucei* RNA editing ligase 1 (REL1) bound to compound V2, a low-micromolar naphthalene-based inhibitor [25]. REL1 is essential for the survival of the unicellular parasite *Trypanosoma brucei* [26]. It is a component of the parasitic editosome that religates the RNA nicks caused by extensive uridylate insertions and deletions [27–31]. REL1 has a deep ATP-binding pocket where compound V2 likely binds [25].

**Fig 4:**
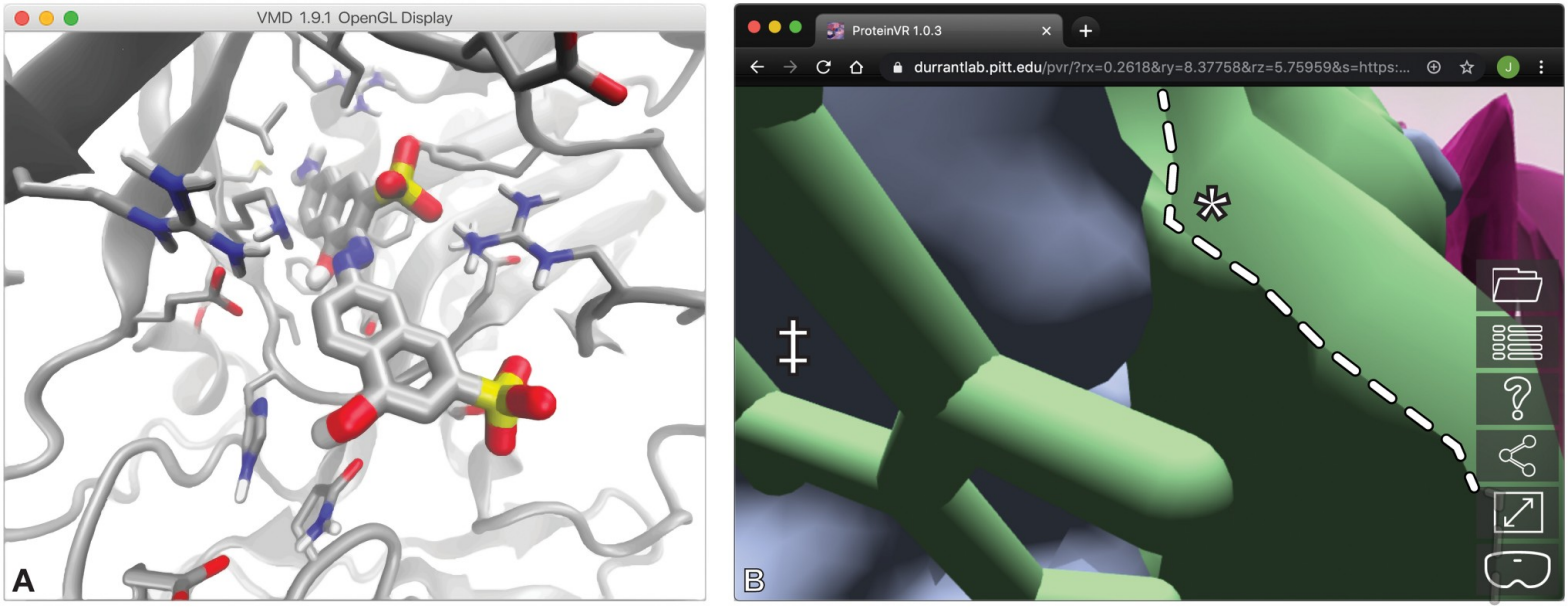
Two examples that show the advantages of ProteinVR use. A) An illustration of compound V2 docked into the REL1 ATP-binding pocket, visualized using VMD. Despite the use of fog and perspective view, perceiving critical protein/ligand interactions without true stereoscopic 3D is at times challenging. B) An illustration of the space between open- and closed-pocket LARP1 surfaces, visualized using ProteinVR in non-VR mode. Prior to entering VR mode, camera-adjacent sections of the surfaces are often clipped (highlighted with a white dotted line), as is typical of non-VR programs. Clipping is easier to avoid in VR because the camera position is finely controlled by simple head movements, and VR provides a wider field of view.

We used Gypsum-DL [32], MGLTools (http://mgltools.scripps.edu/), and AutoDock Vina [33] to dock a model of V2 into the REL1 pocket. We then used VMD [2] and ProteinVR to separately examine the protein-ligand interactions characteristic of the top Vina-predicted pose. To make the comparison as fair as possible, we used fog and perspective view in VMD to simulate depth (Fig 4A). Even so, it was at times challenging to perceive the critical interactions. Because the REL1 pocket is so deep, closer atoms often pass in front of more buried features, leading to some confusion in the absence of true depth perception. Non-VR programs such as VMD and PyMOL [3] cannot provide the same natural-feeling experience that is typical of observing objects in the physical world.

Viewing the same protein/ligand complex in ProteinVR with an HTC Vive VR headset was more intuitive. The molecular structure had presence. With almost no effort, the tester was able to see that the two phosphate groups of the ligand flank a positively charged arginine residue, that other positively charged residues may contribute to molecular recognition via electrostatic interactions, that ligand hydroxyl groups form hydrogen bonds with the protein, etc. It was also immediately clear that the Vina-predicted ligand pose may be incorrect. One of the naphthalene moieties could more optimally participate in a π-π stacking interaction with the protein if rotated slightly, and a buried amino moiety appears to have no hydrogen-bond partner. The ability to look around obstructing atoms with simple head movements, together with true stereoscopic depth perception, made obtaining these kinds of insights particularly intuitive.

This test case illustrates how ProteinVR can benefit both researchers and educators. We have spent many hours analyzing protein/ligand complexes using non-VR programs. These programs are effective, but they require users to trick their brains into a kind of faux depth perception by using fog and perspective view, and by rocking the molecular structures about carefully selected pivot points. Seeing the structures in stereoscopic 3D substantially reduces the cognitive load required to appreciate these interactions, ultimately speeding analysis. Students in particular stand to benefit from the VR approach. Perceiving the 3D geometry of the bound ligand pose becomes intuitive, allowing students to focus on the meaning of the interactions rather than the presentation.

#### Conformations extracted from molecular-dynamics simulations

As a second test case, we used ProteinVR to reexamine a previously published molecular-dynamics (MD) simulation [34]. The simulation captured the dynamics of La-related protein 1 (LARP1), an RNA-binding protein that regulates ribosome production, cell growth, and proliferation. Our previous work showed that a LARP1 pocket known to bind mRNA caps is highly dynamic. Using the POVME algorithm [35, 36], we identified the MD-sampled conformations with both the largest and smallest pocket volumes. This analysis, together with careful examination of the structures in VMD, was critical for characterizing pocket dynamics [34].

Reexamining these pocket conformations with ProteinVR on an HTC Vive VR headset allowed for a far more intuitive analysis. ProteinVR does not yet support MD-trajectory visualization, but it was not difficult to concatenate the largest- and smallest-pocket conformations into a single PDB file and to assign different chain IDs to each. We loaded this two-structure PDB file into ProteinVR and applied a differently colored surface representation to each conformation. To better appreciate the pocket-volume difference, we attempted to position the ProteinVR camera between the surfaces of the smallest-volume (collapsed) and largest-volume (open) pockets.

Before entering VR mode, it was difficult to observe this inter-surface space (i.e., the space corresponding to the pocket-volume difference) from within. As with other non-VR programs, camera-adjacent surface sections were often clipped (Fig 4B). In contrast, appreciating the space was much easier in VR mode. The tester simply used ProteinVR’s teleportation navigation system to move immediately adjacent to the surface of the smallest-volume (collapsed) pocket. By pushing his head forward through this outer surface, he was able to easily examine the size and contours of the space between the two surfaces. This type of experience cannot replace objective numerical analyses such as those that POVME provides, but it certainly can yield insights to guide future research. The tester himself, despite extensive experience with both VR and LARP1, had a gleeful “eureka” moment when first observing the pocket-volume difference in VR.

This test case similarly illustrates how ProteinVR can benefit both researchers and educators. Rational ligand design often requires a thorough appreciation of the 3D volume that a binding pocket occupies. But visualizing narrow and/or buried pockets is sometimes difficult without VR. In contrast, ProteinVR in VR mode enables minute movements by tracking head motions. Both researchers and students can easily avoid clipping while looking about pocket interiors. The stereoscopic vision possible in VR also give a better sense of the pocket dimensions.

### Comparison with other programs

#### Molecule-to-mesh pipelines

ProteinVR makes setting up VR molecular visualizations particularly easy. In contrast, some other VR programs rely on complex software pipelines that require users to install (and master) third-party modeling programs such as Blender (Blender Foundation) and Unity (Unity Technologies). Users must setup molecular representations (e.g., ribbon, stick, surface) during the initial modeling stage, making it impossible to change the representation in real-time VR.

The open-source BlendMol [37] plugin for Blender [38] is one example of this effective but difficult-to-manage approach. BlendMol/Blender can produce photorealistic images of protein structures that are well suited for scientific publication and educational outreach. Third-party Blender plugins can also export BlendMol models to VR-compatible formats. But the BlendMol method for preparing VR models is far from automated and requires some expertise in 3D modeling.

RealityConvert [39], like BlendMol, provides a molecule-to-mesh pipeline that generates molecular meshes for VR and AR scenes. An easy-to-use web app helps overcome some barriers to use. But the web app only accepts very small molecules (< 200 lines). Processing larger molecules requires the command-line version and its four dependencies: PyMOL [3], Blender [38], Open Babel [40], and Molconvert (ChemAxon). Many other VR and AR approaches for molecular visualization involve similarly challenging software pipelines [19, 21, 41]. In contrast, ProteinVR requires no download or dependencies and so is more accessible.

#### Desktop applications

A number of desktop applications enable VR molecular visualization directly, without requiring a complex pipeline. These desktop programs often limit their compatibility to high-end VR devices [7, 11, 41, 42]. In contrast, ProteinVR is generally more accessible because it supports a broad range of VR headsets as well as non-VR fallback approaches such as device-orientation-based viewing. This broad support is possible because ProteinVR relies on the WebVR API, which standardizes the way VR-enabled websites interact with various devices, as well as the BabylonJS JavaScript game engine, which provides a broad range of video-game-style navigation schemes. As a web-based app, ProteinVR also requires no download or installation, further improving accessibility.

That having been said, desktop programs that cater to high-end VR headsets are able to implement useful features that ProteinVR currently lacks. Molecular Rift [7] is a good example of such a desktop program. This innovative, open-source VR application allows users to navigate molecular structures without VR controllers, using hand gestures. The commercial program Nanome (Nanome Inc.) is a second notable example. Nanome’s easy-to-use and detailed user interface permits not only molecular visualization, but also molecular manipulation (e.g., *in silico* mutagenesis). The free version of Nanome does come with some important limitations, however. For example, VR molecular scenes created with the free version are entirely public. In fact, as we were testing Nanome, another user joined our room and was able to observe our activities.

#### Other web applications

Recognizing the advantages of the web-based approach, others have also explored online VR molecular-visualization systems [20, 43]. One example is iview [20]. Though the iview website includes a “virtual reality” button, this button was not functional on any of the browsers we tested. The iview source code does make reference to WebVR, so perhaps it is the user interface, rather than the underlying codebase, that is broken. We note also that the iview server went offline after our initial tests, though the connectivity problem may be temporary. Regardless, ProteinVR provides additional features—including 3D environments and device-orientation mode—that iview and other programs currently lack.

#### Unity game engine

The Unity game engine (Unity Technologies) warrants specific mention because it powers several desktop VR applications, including Molecular Rift [7], described above, and Molecular Zoo [10], a program for teaching young students about biomolecules. The open-source library UnityMol [44] even enables on-the-fly molecular-mesh generation in Unity apps, much as 3Dmol.js does for ProteinVR.

Unity has several advantages over the BabylonJS game engine behind ProteinVR. Its advanced editor greatly simplifies development, and its online community has developed many add-ons (both free and commercial) that allow developers to easily add the specific features that their application requires. If desktop Unity applications are properly optimized, their performance also surpasses that of any web-based app because most browsers cap graphics updates at 60 frames per second. Finally, because the WebVR standard is still evolving, Unity applications are arguably more stable, at least for the time being.

Despite these advantages, we built ProteinVR using BabylonJS because it is particularly well suited for web apps. Unity applications can be compiled to run in the browser, but they are almost always far larger than the equivalent BabylonJS app, requiring more time and bandwidth to download. Unity also lacks official support for browser-based apps on mobile devices, and the BabylonJS approach to WebVR is much more straightforward than its Unity counterpart. Because BabylonJS is itself written in JavaScript, integration with web technologies such as WebRTC and the HTML5 DOM (e.g., buttons, popups, menus, etc.) is also much easier. Finally, the Unity engine is closed source, and free use requires Unity-specific branding.

## Availability

We release ProteinVR under the open-source BSD-3-Clause license. The source code can be downloaded anonymously from our public repository at http://durrantlab.com/protein-vr/. The accompanying documentation includes instructions for using and compiling the software. Most users will prefer to simply access the working version we have posted at http://durrantlab.com/pvr/. Users may also report bugs or other issues via the online forum at http://durrantlab.com/forums/forum/protein-vr/.

## Future directions

### Features

ProteinVR’s strength lies in its simplicity. We are eager to avoid “feature creep,” wherein excessive software updates add only minor benefit while complicating use. Other programs exist for model building and analysis. ProteinVR is meant to be a molecular viewer. Future improvements will further enhance that core functionality.

For example, several related programs display molecules using AR. Unlike VR, AR positions virtual objects in the context of the actual world (e.g., virtual protein structures digitally superimposed on an actual, physical table). Some studies suggest that AR helps mitigate VR sickness [24, 45, 46], so AR may be more accessible to some users. On the other hand, to the best of our knowledge, current advanced AR approaches for molecular visualization work only with the expensive Microsoft HoloLens headset and require users to download separate software to prepare the molecular models [19, 21]. It may be that ProteinVR’s 3D environments, teleportation navigation system, and device-orientation/desktop modes will allow even sensitive users to avoid VR sickness. If not, future versions of ProteinVR will incorporate AR viewing. The BabylonJS game engine that powers ProteinVR has preliminary AR support that will likely expand in the coming years.

One desktop VR program called NarupaXR allows users to interact with real-time atomic-resolution MD simulations running on remote computer resources [11, 47–49]. This interactive approach is remarkably intuitive. Unfortunately, because ProteinVR is web based, it cannot interact with a local server streaming MD data to the viewer. Enabling interactive MD would thus require us to maintain a public server backed by the substantial remote computer infrastructure required to run MD simulations. But we are certainly interested in enabling non-interactive MD-trajectory visualization in future versions of ProteinVR. We have already published two browser-based tools for MD-trajectory playback in a non-VR context [50, 51]. Incorporating components of these programs into future versions of ProteinVR could be a productive future direction.

Additional planned features include 1) improved chain and residue labeling, 2) automatic detection and visualization of protein/ligand interactions via our BINANA algorithm [52], and 3) expanded methods for loading molecular data.

### Further testing

Further user testing will evaluate how best to deploy ProteinVR in research and educational settings. Continued use in our lab–together with feedback from our users–will help us further evaluate ProteinVR’s utility in a research context. Members of well funded research labs with dedicated space for VR visualization may prefer desktop applications running on powerful computers with high-end VR headsets. These programs offer an impressive array of features that are difficult to deliver over the web. But in many situations, ProteinVR’s convenience (e.g., compatibility with mobile VR headsets such as the Oculus Quest, easy web access, etc.) may make it the preferred choice.

We expect that ProteinVR’s portability, broad hardware compatibility, and ease of deployment will be particularly useful in an educational context. Lecturers are rarely able to bring an expensive gaming computer with a high-end VR headset to class. And even when such a system is available, it cannot reasonably accommodate large classrooms. In contrast, purchasing low-end VR headsets (e.g., Google Cardboard) so students can view VR scenes on their mobile devices is feasible. A limited number of medium-quality headsets (e.g., the Oculus Quest) can then supplement the mobile-based devices.

Published studies have shown that VR enhances science education generally [53] and chemistry education specifically [54]. We are currently designing a study that will evaluate how ProteinVR improves learning outcomes in both high-school and undergraduate classrooms.

## Supporting information

S1 Video A video that illustrates ProteinVR’s basic features. (MP4)

S1 Fig A flowchart diagram representing the main steps of the ProteinVR algorithm. (PDF)

S1 Table A few of the devices used to test ProteinVR in VR mode, together with the associated hardware specifications. (PDF)
