# Supplementary material for: ProteinVR: Web-based molecular visualization in virtual reality
Source: PLoS Comput Biol. 2020 Mar 31;16(3):e1007747. doi: 10.1371/journal.pcbi.1007747 (PMC7147804; doi:10.1371/journal.pcbi.1007747)
Supplement: S1 Fig — (PDF) [file pcbi.1007747.s002.pdf]

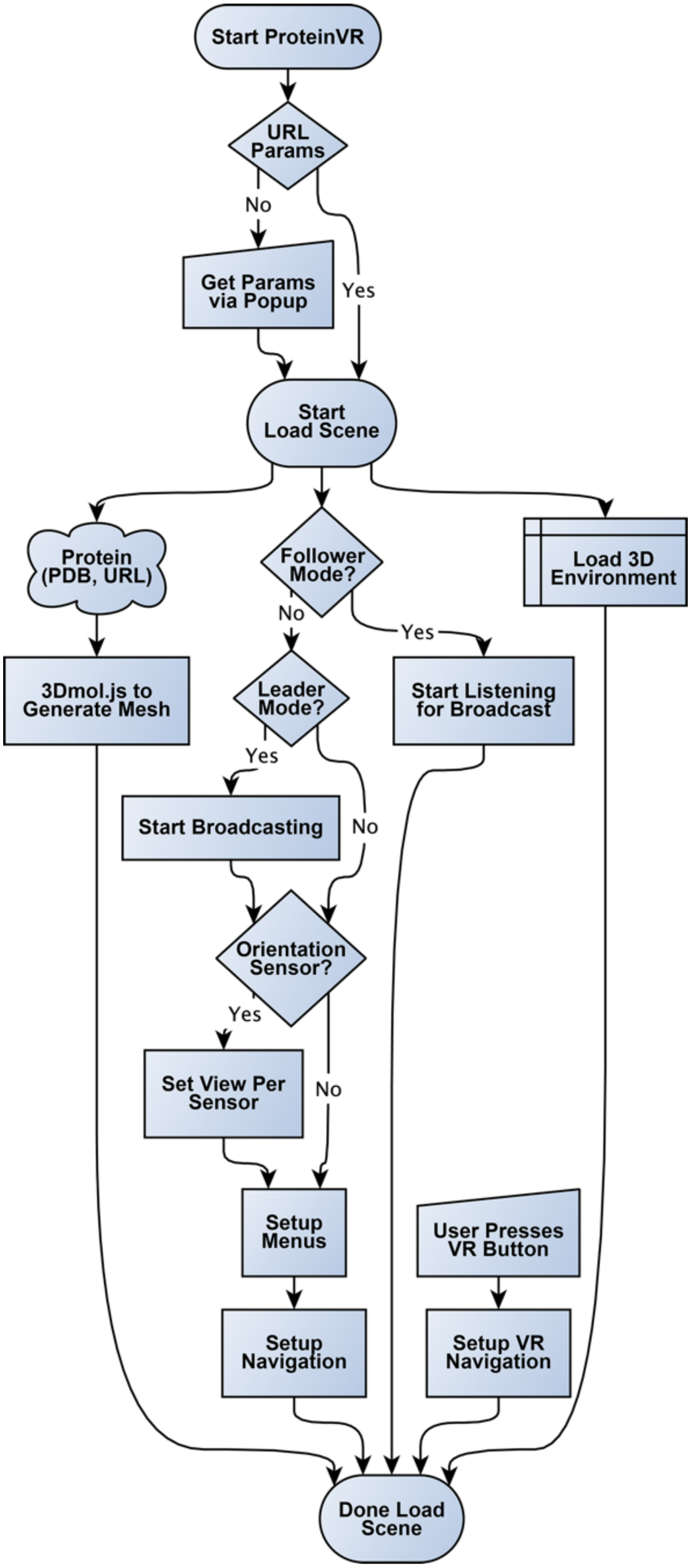

S1 Fig. Flowchart diagram representing the main steps of the ProteinVR algorithm. Oval nodes representing starting and stopping points. Diamonds represent decision points. Trapezoids represent points of manual input. The cloud represents data loaded from a remote source. The partitioned rectangle represents data loaded from within the ProteinVR app itself (internal storage). Plain rectangles represent algorithmic process steps.
