# Supplementary material for: ProteinVR: Web-based molecular visualization in virtual reality
Source: PLoS Comput Biol. 2020 Mar 31;16(3):e1007747. doi: 10.1371/journal.pcbi.1007747 (PMC7147804; doi:10.1371/journal.pcbi.1007747)
Supplement: S1 Table — (PDF) [file pcbi.1007747.s003.pdf]

S1 Table. A few of the devices used to test ProteinVR in VR mode, together with the associated hardware specifications.

Device Type	Brand	CPU(s)	GPU(s)	Memory	OS
**Desktop**	Dell Precision 3630 Tower (R)	Intel Core (TM) i7-8700K @ 3.7 GHz, 6 Cores, 12 Threads	NVIDIA GeForce GTX 1080, Intel (R) UHD Graphics 630	16 GB	Microsoft Windows 10 Pro
**Mobile**	Samsung Galaxy S10 SM-G973U	Qualcomm Snapdragon 855: Kryo 485 Dual-Core @ 2.8 GHz, Kryo 485 Dual-Core @ 2.4 GHz, and Kryo 485 Quad-Core @ 1.7 GHz	Adreno 640	8 GB	Android 10
**Mobile**	iPhone X	Apple A11 Bionic Hexa-core @ 2.39 GHz	Apple-Designed 3 Core	3 GB	iOS 11

ProteinVR is designed to run on a broad range of devices. The information presented in this table should give users a sense for the hardware required, but many lower-powered devices can also run ProteinVR successfully. Using ProteinVR in non-VR mode is even less computationally demanding. We have intentionally omitted VR-specific devices such as the Oculus Quest, as these obviously have the hardware required to run VR.
